# Structural Insight into the Rotational Switching Mechanism of the Bacterial Flagellar Motor

**DOI:** 10.1371/journal.pbio.1000616

**Published:** 2011-05-10

**Authors:** Tohru Minamino, Katsumi Imada, Miki Kinoshita, Shuichi Nakamura, Yusuke V. Morimoto, Keiichi Namba

**Affiliations:** 1 Graduate School of Frontier Biosciences, Osaka University, Osaka, Japan; 2 PRESTO, JST, Saitama, Japan; 3 Department of Macromolecular Science, Osaka University, Osaka, Japan; Cambridge University, United Kingdom

## Abstract

The bacterial flagellar motor can rotate either clockwise (CW) or counterclockwise (CCW). Three flagellar proteins, FliG, FliM, and FliN, are required for rapid switching between the CW and CCW directions. Switching is achieved by a conformational change in FliG induced by the binding of a chemotaxis signaling protein, phospho-CheY, to FliM and FliN. FliG consists of three domains, FliG_N_, FliG_M_, and FliG_C_, and forms a ring on the cytoplasmic face of the MS ring of the flagellar basal body. Crystal structures have been reported for the FliG_MC_ domains of *Thermotoga maritima*, which consist of the FliG_M_ and FliG_C_ domains and a helix E that connects these two domains, and full-length FliG of *Aquifex aeolicus*. However, the basis for the switching mechanism is based only on previously obtained genetic data and is hence rather indirect. We characterized a CW-biased mutant (*fliG*(ΔPAA)) of *Salmonella enterica* by direct observation of rotation of a single motor at high temporal and spatial resolution. We also determined the crystal structure of the FliG_MC_ domains of an equivalent deletion mutant variant of *T. maritima* (*fliG*(ΔPEV)). The FliG(ΔPAA) motor produced torque at wild-type levels under a wide range of external load conditions. The wild-type motors rotated exclusively in the CCW direction under our experimental conditions, whereas the mutant motors rotated only in the CW direction. This result suggests that wild-type FliG is more stable in the CCW state than in the CW state, whereas FliG(ΔPAA) is more stable in the CW state than in the CCW state. The structure of the TM-FliG_MC_(ΔPEV) revealed that extremely CW-biased rotation was caused by a conformational change in helix E. Although the arrangement of FliG_C_ relative to FliG_M_ in a single molecule was different among the three crystals, a conserved FliG_M_-FliG_C_ unit was observed in all three of them. We suggest that the conserved FliG_M_-FliG_C_ unit is the basic functional element in the rotor ring and that the PAA deletion induces a conformational change in a hinge-loop between FliG_M_ and helix E to achieve the CW state of the FliG ring. We also propose a novel model for the arrangement of FliG subunits within the motor. The model is in agreement with the previous mutational and cross-linking experiments and explains the cooperative switching mechanism of the flagellar motor.

## Abstract 2

Structural analysis of a clockwise-biased rotation mutant of the bacterial flagellar rotor protein FliG provides a new model for the arrangement of FliG subunits in the motor, and novel insights into rotation switching.

## Author Summary

The bacterial flagellum is a rotating organelle that governs cell motility. At the base of each flagellum is a motor powered by the electrochemical potential difference of specific ions across the cytoplasmic membrane. In response to environmental stimuli, rotation of the motor switches between counterclockwise and clockwise, with a corresponding effect on the swimming direction of the cell. Switching is triggered by the binding of the signaling protein phospho-CheY to FliM and FliN, and achieved by conformational changes in the rotor protein FliG. The actual switching mechanism, however, remains unclear. In this study, we characterized a *fliG* mutant of *Salmonella* that shows an extreme clockwise-biased rotation, and determined the structure of a fragment of FliG (FliG_MC_) of the equivalent mutant variant of *Thermotoga maritima*. FliG_MC_ is composed of two domains and covers the regions essential for torque generation and FliM binding. We showed that the mutant structure has a conformational change in the helix connecting the two domains, leading to a domain orientation distinct from that of the wild-type FliG. On the basis of this structure, we propose a new model for the arrangement of FliG subunits in the rotor that is consistent with the previous mutational studies and explains how cooperative switching occurs in the motor.

## Introduction

Bacteria such as *Escherichia coli* and *Salmonella enterica* swim by rotating multiple flagella, which arise randomly over the cell surface. Each flagellum is a huge protein complex made up of about 30 different proteins and can be divided into three distinct parts: the basal body, the hook, and the filament. The basal body is embedded in the cell envelope and acts as a reversible motor powered by a proton motive force across the cytoplasmic membrane. The hook and the filament extend outwards in the cell exterior. The filament is a helical propeller that propels the cell body. The hook connects the basal body with the filament and functions as a universal joint to transmit torque produced by the motor to the filament. The flagellar motor can exist in either a counterclockwise (CCW) or clockwise (CW) rotational state. CCW rotation causes the cell to swim smoothly in what is termed a run, whereas brief CW rotation of one or more flagella causes a tumble. The direction of motor rotation is controlled by environmental signals that are processed by a sensory signal transduction pathway to generate chemotaxis behavior [1]–[3].

Five flagellar proteins, MotA, MotB, FliG, FliM, and FliN, are involved in torque generation. Two integral membrane proteins, MotA and MotB, form the stator, which converts an inwardly directed flux of H^+^ ions through a proton-conducting channel into the mechanical work required for motor rotation. The FliG, FliM, and FliN proteins form the C ring on the cytoplasmic side of the MS ring, which is assembled from 26 subunits of a single protein, FliF, and this complex acts as the rotor of the flagellar motor [1]–[3]. An electrostatic interaction between the cytoplasmic loop of MotA and FliG is thought to be involved in torque generation [4],[5] and in stator assembly around the rotor [6]. The protonation-deprotonation cycle of a highly conserved aspartic acid residue in MotB is coupled to the movement of the MotA cytoplasmic loop to generate torque [7]–[9].

Because FliG, FliM, and FliN are also responsible for switching the direction of motor rotation, their assembly is called the switch complex [10]. Binding of a chemotactic signaling protein CheY-phosphate (CheY-P) to FliM and FliN is presumed to induce conformational changes in FliG that result in a conformational rearrangement of the rotor-stator interface, allowing the motor to spin in the CW direction [11],[12]. The switching probability is also affected by motor torque, suggesting that the switch complex senses the stator-rotor interaction as well as the concentration of CheY-P [13],[14]. Recently, turnover of FliM and heterogeneity in the number of FliM subunits within functioning motors have been reported [15],[16]. The turnover rate is increased by the presence of CheY-P, implying that turnover of FliM may be directly involved in the switching process [15].

FliG forms a ring on the cytoplasmic face of the MS ring with 26-fold rotational symmetry [17],[18]. FliG consists of three domains, FliG_N_, FliG_M_, and FliG_C_. FliG_N_ is responsible for association with the cytoplasmic face of the MS ring [17],[19], and FliG_M_ and FliG_C_ are required for an interaction with FliM [20]. The FliG_M_ domains of adjacent subunits are fairly close to each other in the FliG ring [21]. The crystal structure of FliG_MC_ of *Thermotaoga martima* (Tm-FliG_MC_) shows that FliG_M_ and FliG_C_ are connected by an extended α-helical linker (helix E) [22]. The linker contains two well-conserved Gly residues and hence might be flexible [22]. This finding is supported by genetic analyses of FliG and a computer-generated prediction of its secondary structure [23],[24]. Critical charged residues, which are responsible for an interaction with MotA [4]–[6], are clustered together along a prominent ridge on FliG_C_ [25]. It has been shown that the elementary process of torque generation by the stator-rotor interaction is symmetric in CCW and CW rotation [26], although the torque-speed curves are distinct between them [27].

A recent report on the full-length FliG structure of *Aquifex aeolicus* has shown two distinct conformational differences between the full-length FliG and FliG_MC_ structures [28]. The helix E linker is held in a closed conformation by packing tightly against an α-helix (helix n), which connects FliG_N_ to FliG_M_ in a way similar as helix E connects FliG_M_ and FliG_C_ in the full-length FliG structure. Helix E is dissociated from FliG_M_ in the Tm-FliG_MC_ structure, resulting in its being in an open conformation. The conformation of FliG_C_ is also different in these two structures. Combined with the previous genetic data, it has been proposed that the closed conformation represents FliG during CCW rotation and that switching to CW rotation may be accompanied by the dissociation of helix E from FliG_M_ to form an open conformation.

The *S. enterica* FliG(ΔPAA) mutant protein has three-amino-acid deletion at positions 169 to 171. Motors containing this protein are extremely CW biased [29]. The mutant motors remain in CW rotation even in the presence of a *cheY* deletion, indicating that the motor is locked in the CW state [29]. Therefore, it is likely that binding of CheY-P to FliM may introduce a conformational change in FliG similar to the one introduced by the in-frame PAA deletion. To elucidate the switching mechanism, we crystallized a fragment of a *T. maritima* FliG mutant variant, FliG_MC_(ΔPEV), which contains a deletion equivalent to *S. enterica* FliG_MC_(ΔPAA), and determined its structure at 2.3 Å resolution. Based on the structural difference among full-length *A. aeolicus* FliG, wild-type Tm-FliG_MC_, and its deletion variant, we suggest that a reorientation of helix E relative to FliG_M_ is important for switching and propose a new model for the arrangement of FliG subunits in the motor.

## Results

### Characterization of *S. enterica fliG*(ΔPAA) Mutant

The motors of the *fliG*(ΔPAA) mutant rotated only CW (Figure S1A), whereas wild-type motors rotated exclusively CCW under our experimental conditions. The motors of the deletion mutant produced normal torque under a wide range of external-load conditions, indicating that the deletion does not affect the torque generation step (Figure S1B). Introduction of a *cheA-Z* deletion, which causes wild-type motors to spin exclusively CCW [30], into the *fliG*(ΔPAA) mutant did not change the CW-locked behavior. These results are in good agreement with a previous report [29].

**Figure 1:**
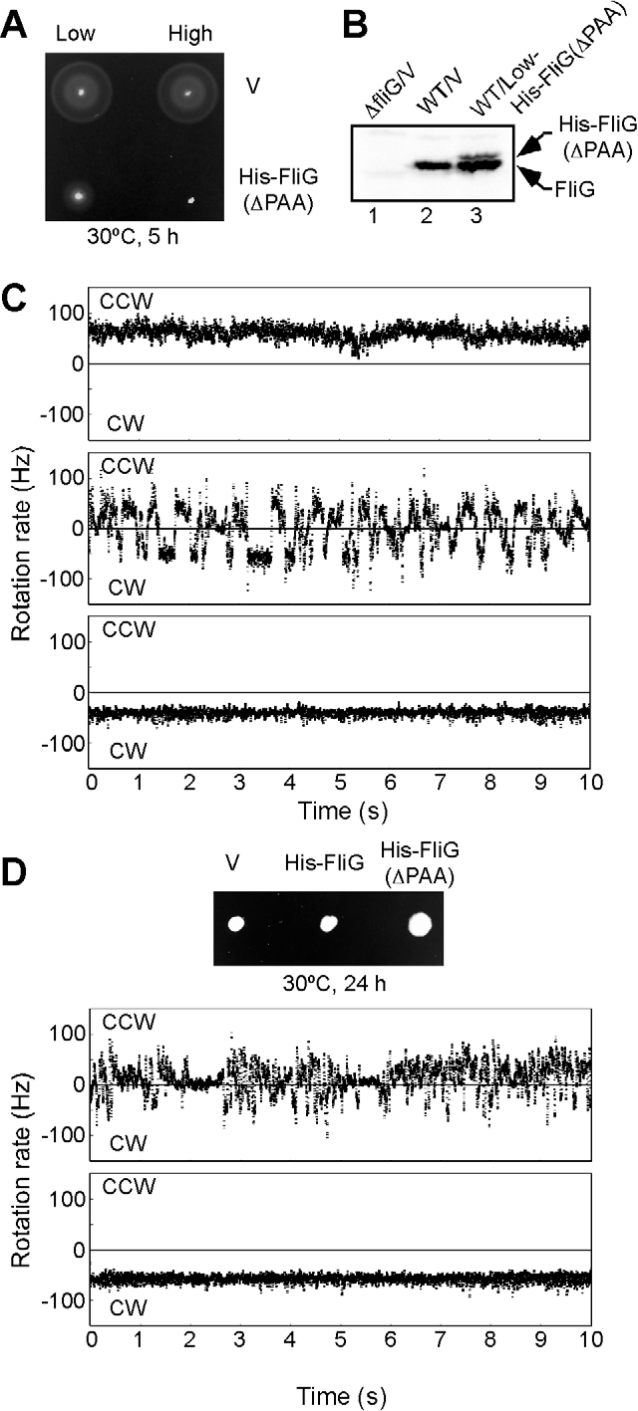
Dominant-negative effect of FliG(ΔPAA) on motility of wild-type cells. (A) Motility of SJW1103 cells (wild-type) transformed with pET19b (indicated as Low-V), pTrc99A (indicated as High-V), pGMK4000 (pET19b/His-FliG(ΔPAA), indicated as Low-FliG(ΔPAA)), and pGMM4500 (pTrc99A/His-FliG(ΔPAA), indicated as High-FliG(ΔPAA)) in semi-solid agar plates. (B) Expression levels of FliG and His-FliG(ΔPAA). Immunoblotting, using polyclonal anti-FliG antibody, of whole cell proteins. Lane 1, MKM1/pET19b (indicated as Δ*fliG*/V); lane 2, SJW1103/pET19b (indicated as WT/V); lane 3, SJW1103/pGMK4000 (indicated as WT/Low-His-FliG(ΔPAA)). Arrows indicate positions of FliG and His-FliG(ΔPAA). (C) Measurement of CCW and CW rotation of the flagellar motor by bead assays. We used SJW46 (*fliC*(Δ204–292)) as a host because it produces flagellar motors with the sticky flagellar filaments, which are easily labeled with polystyrene beads. CCW, counterclockwise rotation; CW, clockwise rotation. Upper panel: SJW46 carrying pET19b. Middle panel: SJW46 carrying pGMK4000. Bottom panel: SJW46 carrying pGMM4500. (D) Effect of FliG(ΔPAA) on motility of a Δ*cheA-Z* mutant. Upper panel: Motility of SJW3076 (Δ*cheA-Z*) transformed with pET19b, pGMK3000 (pET19b/His-FliG), or pGMK4000 in semi-solid agar. Middle panel: measurement of CCW and CW rotation of the flagellar motor of MM3076iC/pGMK4000. Bottom panel: measurement of CCW and CW rotation of the flagellar motor of MM3076iC/pGMM4500.

Switching between the CW and CCW states is highly cooperative [31]–[34]. The switching mechanism can be explained by a conformational spread model, in which a switching event is mediated by conformational changes in a ring of subunits that spread from subunit to subunit via nearest-neighbor interactions [34],[35]. Therefore we investigated rotation of a single motor composed of wild-type and mutant FliG subunits at different ratios. FliG(ΔPAA) inhibited expansion of wild-type colonies in semi-solid agar (Figure 1A), even when its expression level was ca. 5-fold lower than the level of wild-type FliG expressed from the chromosome (Figure 1B). Bead assays revealed that the decrease in colony expansion results from an increase in both switching frequency and prolonged pausing (Figure 1C). In addition, a low level expression of FliG(ΔPAA) partially increased the colony expansion of the Δ*cheA-Z* smooth-swimming mutant, presumably because switching now occurred (Figure 1D, upper and middle panels). These results suggest that even a small fraction of FliG(ΔPAA) in a motor can affect the CW-CCW switching.

The CW-CCW transition, which is very fast in wild-type motors, became significantly longer in mixed motors (Figure 1), suggesting that, as proposed previously [24], the motor can exist in multiple states. A much higher expression of FliG(ΔPAA) completely inhibited wild-type motility (Figure 1D) and did not increase the colony size of the Δ*cheA-Z* mutant in semi-solid agar plates because of the extreme CW-biased rotation of its flagella (Figure 1C and D, lower panel), in agreement with data showing that a higher expression level of wild-type FliG is required for complementation of the *fliG*(ΔPAA) mutant (Figure S2). Therefore, we conclude that wild-type FliG is more stable in the CCW state than in the CW state, whereas FliG(ΔPAA) is more stable in the CW state than in the CCW state.

### Limited Proteolysis of FliG and FliG(ΔPAA)

**Figure 2:**
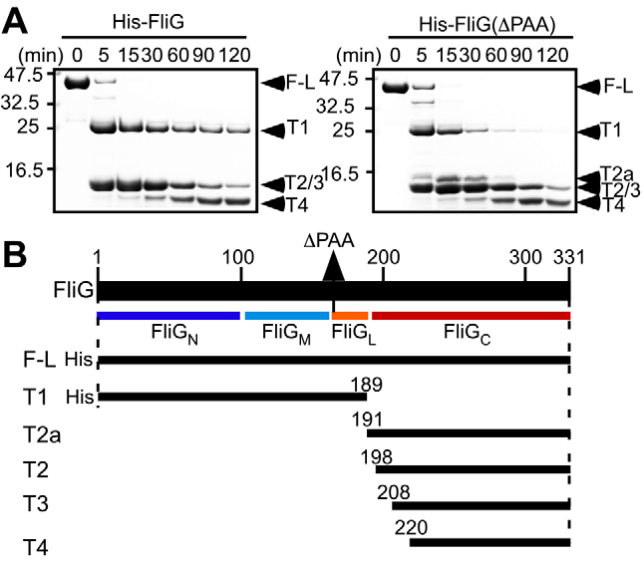
Conformation of FliG in solution. (A) Protease sensitivity of His-FliG (left panel) and His-FliG(ΔPAA) (right panel). Arrowheads indicate intact molecule and proteolytic products on SDS-PAGE gels with labels corresponding to those in the diagram shown in (B). (B) Proteolytic fragments identified by MALDI-TOF mass spectroscopy and N-terminal amino acid sequencing.

To identify structural differences between the CW and CCW states of FliG, we carried out limited trypsin proteolysis of the wild-type and mutant FliG proteins and analyzed the products by matrix-assisted laser desorption ionization time-of-flight (MALDI-TOF) mass spectrometry and N-terminal amino-acid sequencing (Figure 2). Both the wild-type and mutant FliG proteins were cleaved between helix E and FliG_C_, producing the T1 and T2a fragments. This indicates that there is a flexible region between them. The T1 fragment derived from FliG(ΔPAA) was less stable than the T1 fragment from wild-type FliG, suggesting that the deletion causes a conformational change in FliG_M_ and helix E. In contrast, the T2a fragment was more stable in FliG(ΔPAA) than in the wild-type. The T2a fragment derived from the wild-type FliG protein was detected by MALDI-TOF but not on SDS-PAGE gels, indicating that the wild-type T2a fragment is rapidly converted into the T2 fragment. These results suggest that the deletion also influences the conformation in the region between helix E and FliG_C_.

### Structural Comparison of Tm-FliG_MC_ and Tm-FliG_MC_(ΔPEV)

**Figure 3:**
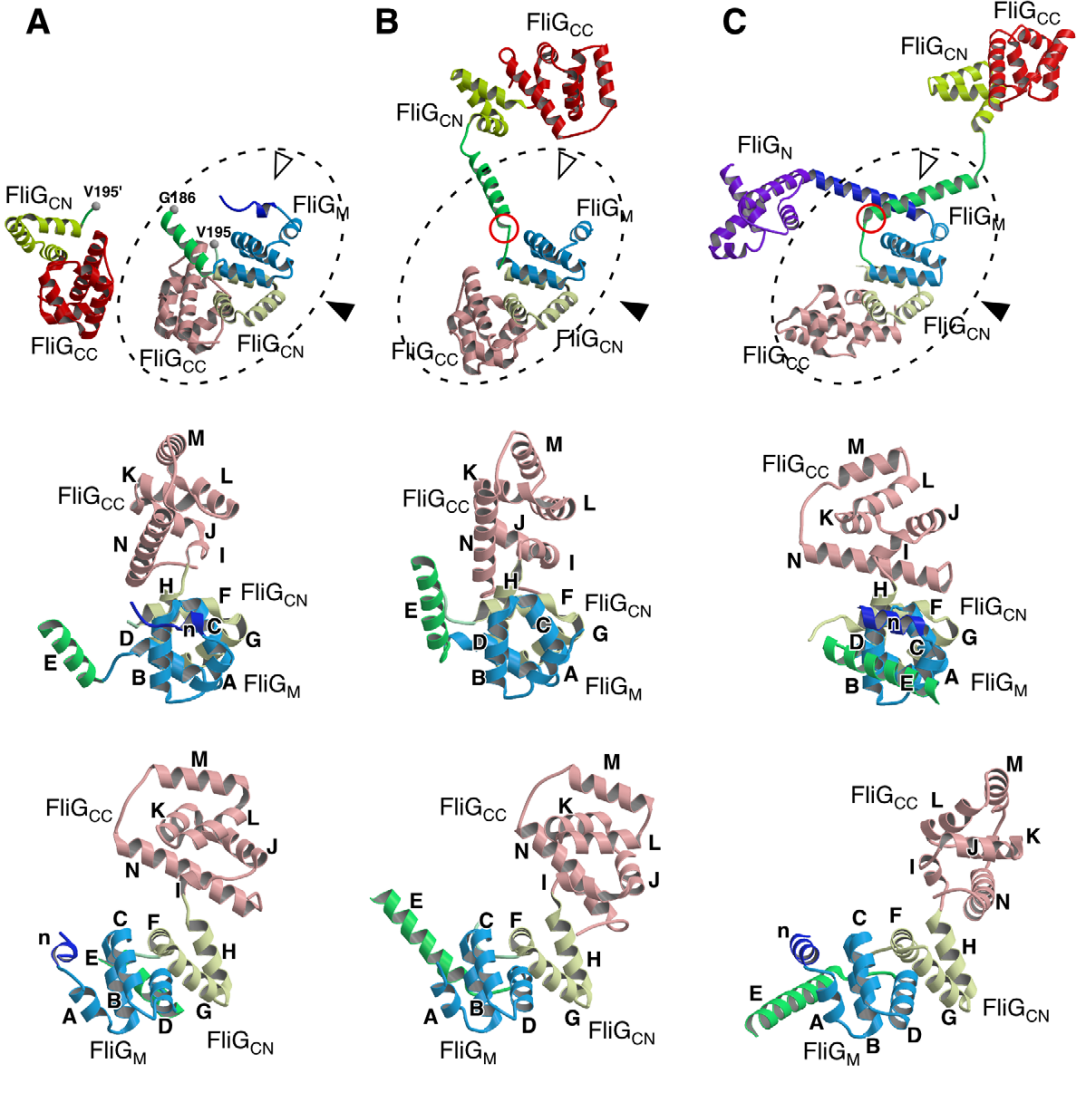
Comparison of the structures of Tm-FliG_MC_(ΔPEV), Tm-FliG_MC_, and Aa-FliG. Cα ribbon representation of (A) Tm-FliG_MC_(ΔPEV), (B) Tm-FliG_MC_ (PDB code 1lkv), and (C) Aa-FliG (PDB code 3hjl), color coded from purple to red going from the N- to the C-terminus. The FliG_M_-FliG_C_ unit with helix E is surrounded by broken line in the upper panels. The white and black arrowheads in the upper panels represent view directions of the middle and the lower panels, respectively. (A, upper panel) Two possible connections between the M-domain and the C-domain (FliG_CN_ and FliG_CC_) in the Tm-FliG_MC_(ΔPEV) crystal are shown. Because the residues between G186 and V195 are invisible in the density map, G186 can be to either V195 or V195'. The two possible C-domains are indicated by vivid and dull colors. (B, C, upper panel) The orientation of the Tm-FliG_MC_ and Aa-FliG molecule is adjusted to that of Tm-FliG_MC_(ΔPEV) by the M-domain (colored cyan). FliG_CN_ and FliG_CC_ of an adjacent molecule related by crystallographic symmetry are shown by dull yellow and dull pink, respectively. The middle panels show comparison of the FliG_M_-FliG_C_ unit structures. All the elements of secondary structure are labeled in alphabetical order from the N- to the C-terminus, except for “n,” which is not found in the Tm-FliG_MC_ structure. The lower panels are viewed from the right of the middle panels.

We tried crystallizing both wild-type FliG and FliG(ΔPAA) from *S. enterica* but did not succeed in obtaining crystals. It has been reported that the crystal structure of a fragment (residues 104–335) of *T. martima* FliG (Tm-FliG_MC_) consists of FliG_M_, FliG_C_, and helix E connecting the two domains ([22]; PDB ID, 1lkv). FliG_C_ can be further divided into two sub-domains (FliG_CN_ and FliG_CC_). Therefore, we introduced the deletion (ΔPEV), equivalent to ΔPAA, into Tm-FliG_MC_ (Tm-FliG_MC_(ΔPEV)) and determined its structure at 2.3 Å resolution by X-ray crystallography (Figure 3).

FliG_M_, FliG_CN_, and FliG_CC_ are composed of five (n, A–D), three (F–H), and six (I–N) helices, respectively (Figure 3). Since the residues between G186 and V195 are invisible in the crystal, there are two possible ways to connect FliG_M_ with FliG_CN_: one is to connect FliG_M_ with its adjacent FliG_CN_ (G186 to V195 in Figure 3A upper panel and Figure S3A), and the other is with a distant FliG_CN_ (G186 to V195' in Figure 3A upper panel and Figure S3A). The Cα distance between G186 and V195, and G186 and V195' is 16.9 Å and 27.9 Å, respectively. Therefore, to connect with the distant FliG_CN_, the invisible chain would have a fully extended conformation. We thus conclude that the connection with the adjacent FliG_CN_ is more plausible.

**Figure 4:**
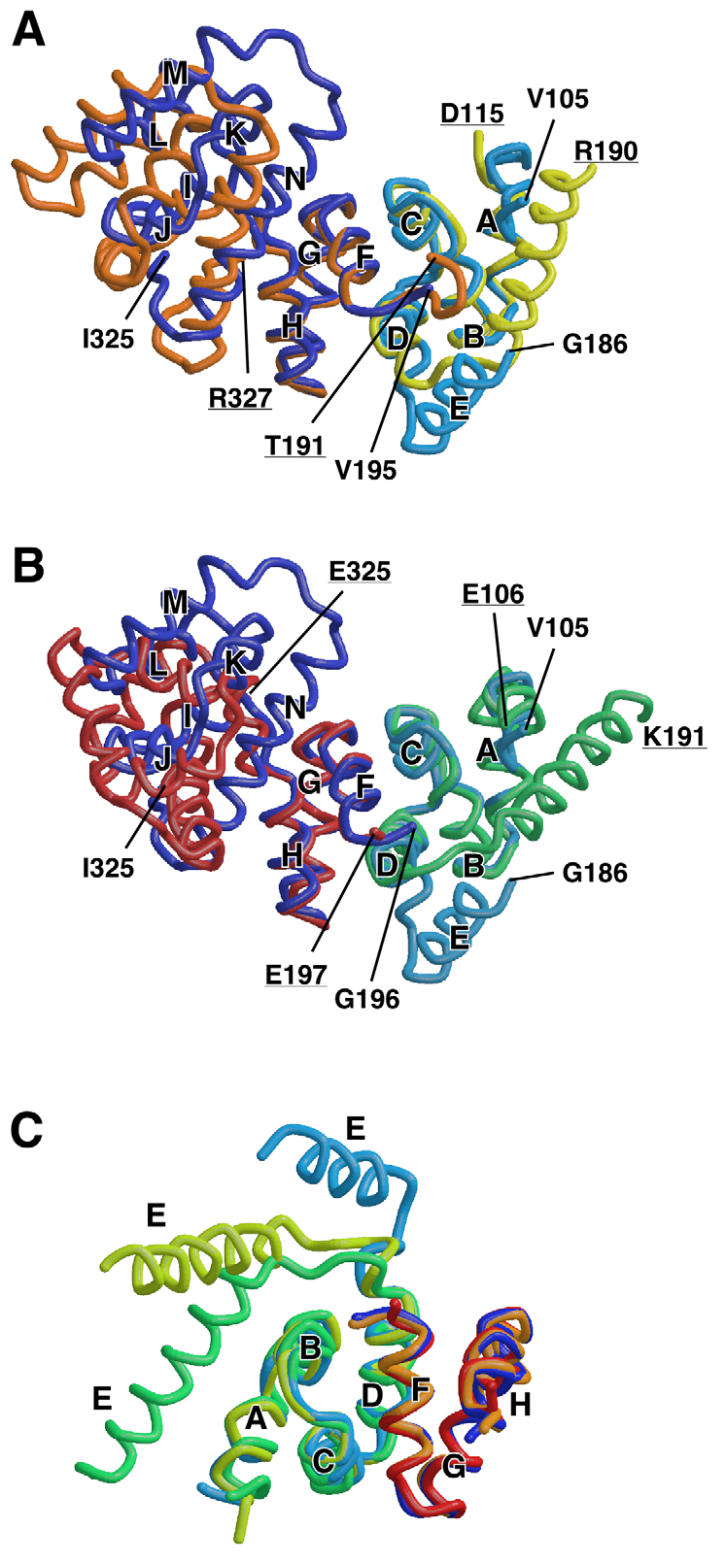
Structural comparison of the FliG_M_-FliG_C_ unit. (A) Comparison of Tm-FliG_MC_(ΔPEV) and wild-type Tm-FliG_MC_ (PDB code 1lkv). A FliG_M_-FliG_C_ unit of wild-type Tm-FliG_MC_, which is composed of FliG_M_ of one subunit and FliG_C_ of the neighboring subunit related by 2-fold crystallographic symmetry, is superimposed onto Tm-FliG_MC_(ΔPEV) using Cα atoms of V117-L165 and G196-F236 for least-square fitting. FliG_M_ with helix E and FliG_C_ of Tm-FliG_MC_(ΔPEV) are colored cyan and blue, respectively. FliG_M_ with helix E and FliG_C_ of wild-type Tm-FliG_MC_ are yellow and orange, respectively. (B) Comparison of Tm-FliG_MC_(ΔPEV) with Aa-FliG (PDB code 3hjl). A FliG_M_-FliG_C_ unit of Aa-FliG, which is composed of FliG_M_ of one molecule and FliG_C_ of the neighboring molecule related by 2-fold crystallographic symmetry, is superimposed onto Tm-FliG_MC_(ΔPEV) using Cα atoms of the same region used in (A). Tm-FliG_MC_(ΔPEV) is shown in the same color as in (A), and FliG_M_ and FliG_C_ of Aa-FliG_MC_ are shown in green and red, respectively. (C) Comparison of the orientation of helix E. The FliG_M_-FliG_CN_ units of wild-type Tm-FliG_MC_ and wild-type Aa-FliG_MC_ are superimposed on Tm-FliG_MC_(ΔPEV). The models are shown in the same colors used in (A) and (B).

Compared with the structure of wild-type Tm-FliG_MC_, FliG(ΔPEV) showed a significant conformational change in the hinge between helix E and FliG_M_, leading to a very different orientation of helix E relative to FliG_M_ (Figure 3A and B, and Figure 4A and C). As a result, some of the residues in FliG_M_ are exposed to solvent in the Tm-FliG_MC_(ΔPEV) structure. This result is in good agreement with the data obtained by limited proteolysis (Figure 2). Thus, the conformational difference in the FliG_M_-helix E hinge between the wild-type and mutant structures may represent the conformational switch between the CW and CCW states of the motor.

The C-terminal half of helix E is disordered and protrudes into the solvent channel in the Tm-FliG_MC_(ΔPEV) crystal (Figure S3A). In contrast, helix E in the wild-type crystal is stabilized by forming an anti-parallel four-helix bundle structure with the E helices of three adjacent subunits related by crystallographic symmetry (Figure S3B) [22]. Therefore, the orientation of FliG_C_ relative to FliG_M_ is different between the wild-type and the deletion variants (Figure 3A and B upper panel). Because the disordered region of helix E is far from the PEV deletion, we conclude that helix E has a highly flexible nature, which may be responsible for the switching mechanism, as suggested before [23],[24].

Tm-FliG_MC_(ΔPEV) also showed a conformational difference in the H–I loop, resulting in a rigid body movement of FliG_CC_ relative to FliG_CN_ (Figure 3A and B middle and lower panels, and Figure 4A). This movement is consistent with the limited proteolysis data because, in the Tm-FliG_MC_(ΔPEV) structure, FliG_CC_ almost covers D199, which is the residue corresponding to R198 in *S. enterica* FliG. It is, however, unclear how the deletion affects the conformation of the H–I loop, because neither direct contact between FliG_CC_ and helix E nor significant structural difference in FliG_CN_ is observed.

### Comparison of the Structure of Tm-FliG_MC_(ΔPEV) with *A. aeolicus* FliG

The crystal structure of full-length *A. aeolicus* FliG (Aa-FliG) showed that the conformation of helix E and the orientation of FliG_CN_ relative to FliG_CC_ are quite distinct from those of wild-type Tm-FliG_MC_ [28]. We compared the Aa-FliG structure with the Tm-FliG_MC_(ΔPEV) structure and found that the conformation of helix E and the relative conformation of FliG_CC_ to FliG_CN_ are also different in those two structures (Figure 3A and C, and Figure 4B and C). The conformational differences are greater than those between Tm- FliG_MC_ and Tm-FliG_MC_(ΔPEV). The conformation of helix E in Aa-FliG seems to be stabilized by interactions of helix E with FliG_M_ and helix n in the crystal (Figure S3C). As mentioned earlier, the conformation of helix E and the orientation of FliG_CC_ to FliG_CN_ are also different between the wild-type and mutant Tm-FliG_MC_ structures. Therefore, these conformational differences among the three structures strongly suggest that both helix E and the linker connecting FliG_CN_ to FliG_CC_ are highly flexible.

### Interaction between FliG_M_ and FliG_CN_

**Figure 5:**
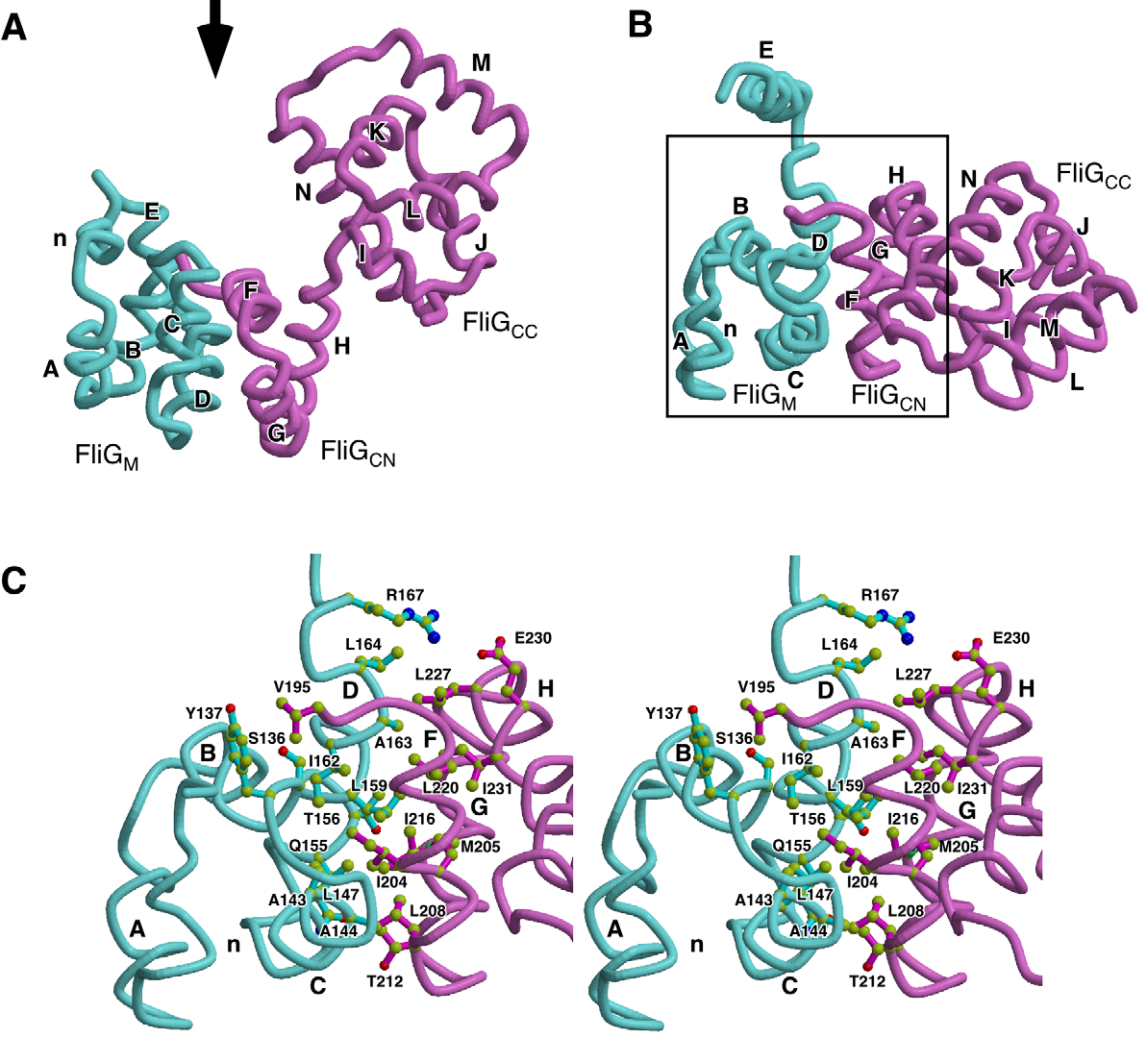
Domain interface between FliG_M_ and FliG_C_. The two domains are colored cyan and magenta, respectively. (A) Structure of Tm-FliG_MC_(ΔPEV). The secondary structure elements are labeled as in Figure 3. (B) Structure of Tm-FliG_MC_(ΔPEV) viewed from the direction of arrow in (A). (C) Stereo view of the domain interface between FliG_M_ and FliG_CN_. The boxed area in (B) is shown. Side chains of the residues contributing strongly to the interaction are shown in a ball-and-stick representation, with carbon, nitrogen, and oxygen atoms indicated by yellow, blue, and red balls, respectively. Bonds are shown with colors of the domains to which they belong.

**Figure 6:**
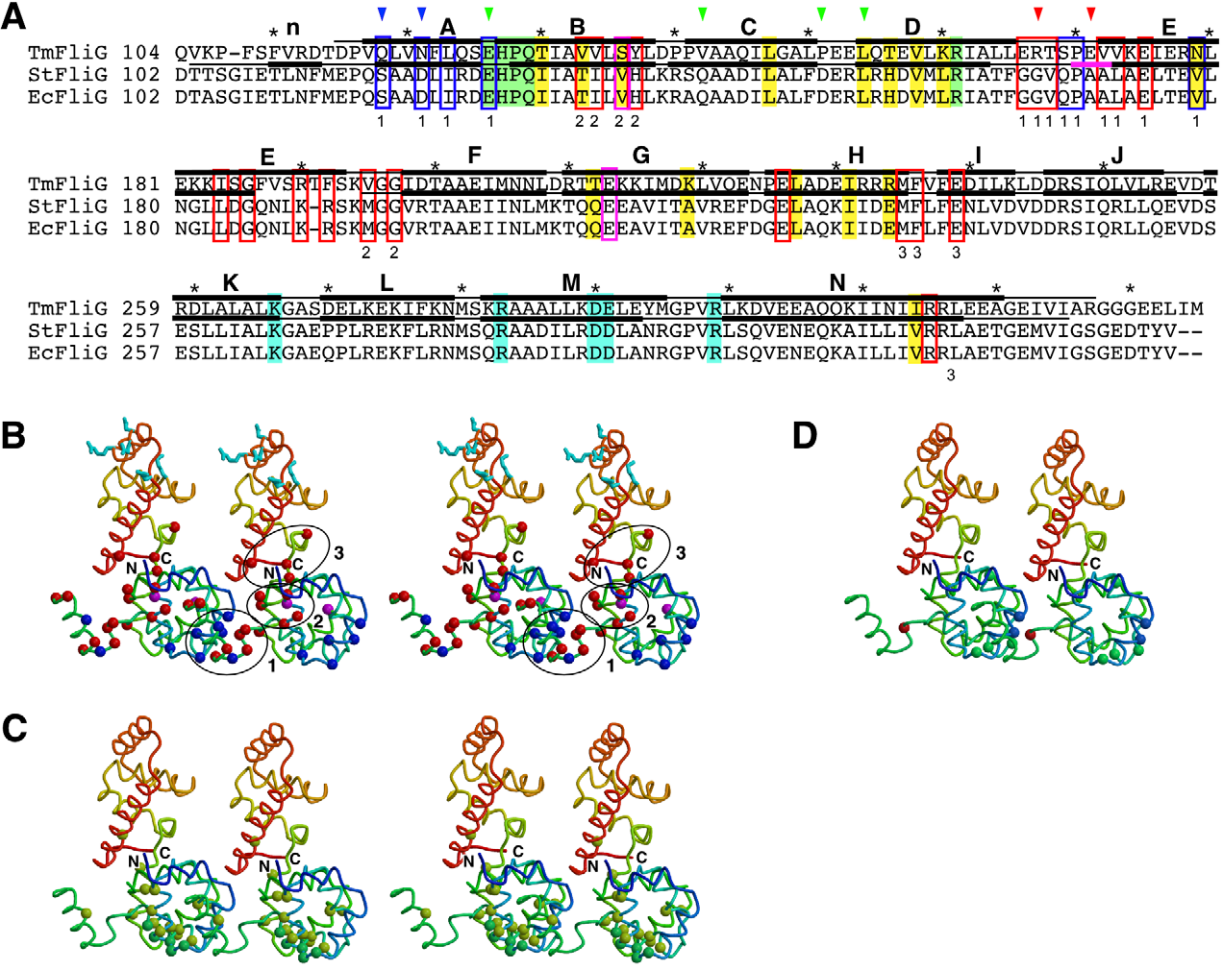
A plausible model for arrangement of FliG subunits in the rotor. (A) A primary sequence alignment of FliG_MC_ from *T. maritima* (TmFliG), *Salmonella* Typhimurium (StFliG), and *Escherichia coli* (EcFliG). The regions involved in the structure models of Tm-FliG_MC_ and Tm-FliG_MC_(ΔPEV) are shown in black bars above and below the Tm-FliG sequence, respectively. The α-helical regions are indicated by thick bars labeled with the same codes used in Figure 3. The region of the three-amino-acid deletion is shown by the magenta bar. The charged residues essential for the motor function are highlighted in cyan. The EHPQR motif is highlighted in green, and the other residues thought to be related to FliM-binding are shaded highlighted in yellow [21],[37]. In vivo cross-linking experiments using various Cys-substitution mutants of FliG_M_ have shown that residues indicated by blue arrows are located near the residues indicated by red ones. The Cys-substitution sites that did not show any cross-linked products are indicated by green arrows [22]. Blue and red boxes indicate point mutations that bias the motor rotation to CCW and CW, respectively [38]. The residues within magenta boxes can give rise to CCW or CW-biased mutants, depending on the substitutions. The numbers under the boxes represent the number of the cluster to which the indicated residues belong. (B–D) Mapping of various mutation sites identified in previous studies on the model of Tm-FliG_MC_(ΔPEV). A stereo pair of the Tm-FliG_MC_(ΔPEV) subunits, color coded from blue to red going from the N- to the C-terminus, is shown in each panel. (B–C) Stereo diagram of the subunit arrangement model. (B) The charged residues essential for motor function are shown in stick representation colored in cyan. Residues at which substitutions affect the direction of motor rotation are indicated by balls: blue, CCW motor bias; red, CW motor bias; magenta, CCW or CW motor bias, depending on the substitution. The clusters of residues targeted by mutations are surrounded by ellipsoids and labeled (1, 2, and 3). (C) Residues involved in FliM binding are indicated by balls: yellow, residues at which substitutions decrease FliM binding; green, the EHPQR motif. (D) Residues substituted with Cys for in vivo cross-linking experiments are shown by balls. Residues indicated in blue cross-linked to residues indicated in red. Residues that produced no cross-linking products are colored in green.

The interaction between FliG_M_ and FliG_CN_, which share the armadillo repeat motif [36] that is often responsible for protein-protein interaction, is very tight in the Tm-FliG_MC_(ΔPEV) crystal, in agreement with a previous report [28]. FliG_M_ and FliG_CN_ can be identified as a single domain, although it is unclear whether the two domains belong to the same molecule or not because the residues between Gly-186 and Val-195 are invisible in the crystal (Figures 3A and S3A). The interaction surface between FliG_M_ and FliG_CN_ is formed by the C-terminal portion of αB, αC, and αD of FliG_M_, and αF, αG, and the N-terminal portion of αH of FliG_CN_, respectively (Figure 5A and B). The interface is highly hydrophobic. Ala-143, Ala-144, Leu-147, Leu-156, Leu-159, Ile-162, and Ala163 of FliG_M_, and Ile-204, Met-205, Leu-208, Ile-216, Leu-220, Leu-227, and Ile-231 of FliG_CN_ are mainly involved in the tight domain interaction. Leu-159 is located at the center of the hydrophobic interface (Figure 5C). Around the hydrophobic core, hydrophilic interactions between Arg-167 and Glu-230, and Gln-155 and Thr-212, also contribute to the domain interaction (Figure 5C). These interactions are also conserved in the wild-type Tm-FliG_MC_ and Aa-FliG crystals, in which FliG_M_ interacts with FliG_CN_ of an adjacent molecule related by crystallographic symmetry (Figures 3 and S3B). The FliG_M_-FliG_CN_ unit in the wild-type Tm-FliG_MC_ structure can be superimposed onto that in Tm-FliG_MC_(ΔPEV) with root mean square deviation of 0.46 Å for corresponding Cα atoms (Figure 4A and C), and that in Aa-FliG with 0.79 Å (Figure 4B and C). These observations support the idea that the FliG_M_-FliG_CN_ unit is a functionally relevant structure [28]. This is in good agreement with the previous mutational study showing that most of the known point mutations that affect FliM-binding [37] are located either on the bottom surface of the FliG_M_-FliG_CN_ unit or on the interaction surface between FliG_M_ and FliG_CN_ (Figure 6A and C).

## Discussion

The default direction of the wild-type flagellar motor of *Salmonella enterica* is CCW, and the binding of CheY-P to FliM and FliN increases the probability of CW rotation. CheY-P binding induces conformational changes in FliM and FliN that are presumably transmitted to FliG, which directly interacts with MotA to produce torque [1],[2]. Mutations located in and around helix E FliG, which connects the FliG_M_ and FliG_C_ domains, generate a diversity of phenotype, including motors that are strongly CW biased, infrequent switchers, rapid switchers, and transiently or permanently paused, suggesting that helix E is directly involved in the switching of the flagellar motor [24]. However, it remains unclear how helix E affects the switch.

To investigate the switching mechanism, we characterized an extreme CW-biased *S. enterica* mutant in which an in-frame deletion of three residues, Pro-169, Ala-170, and Ala-171, in FliG caused an extreme CW-biased rotation even in the absence of CheY. Motors containing the FliG(ΔPAA) protein showed normal torque generation under a wide range of external-load conditions (Figure 1 and Figure 1S). Thus, the conformational change in FliG induced by ΔPAA is presumably similar to one induced by CheY-P binding to FliM and FliN. Limited proteolysis revealed that ΔPAA induces conformational changes in the hinge between FliG_M_ and helix E (Figure 2). This result is in agreement with the crystal structure of Tm-FliG_MC_(ΔPEV), which shows that the orientation of helix E relative to FliG_M_ has changed significantly compared to wild-type FliG (Figure 3).

**Figure 7:**
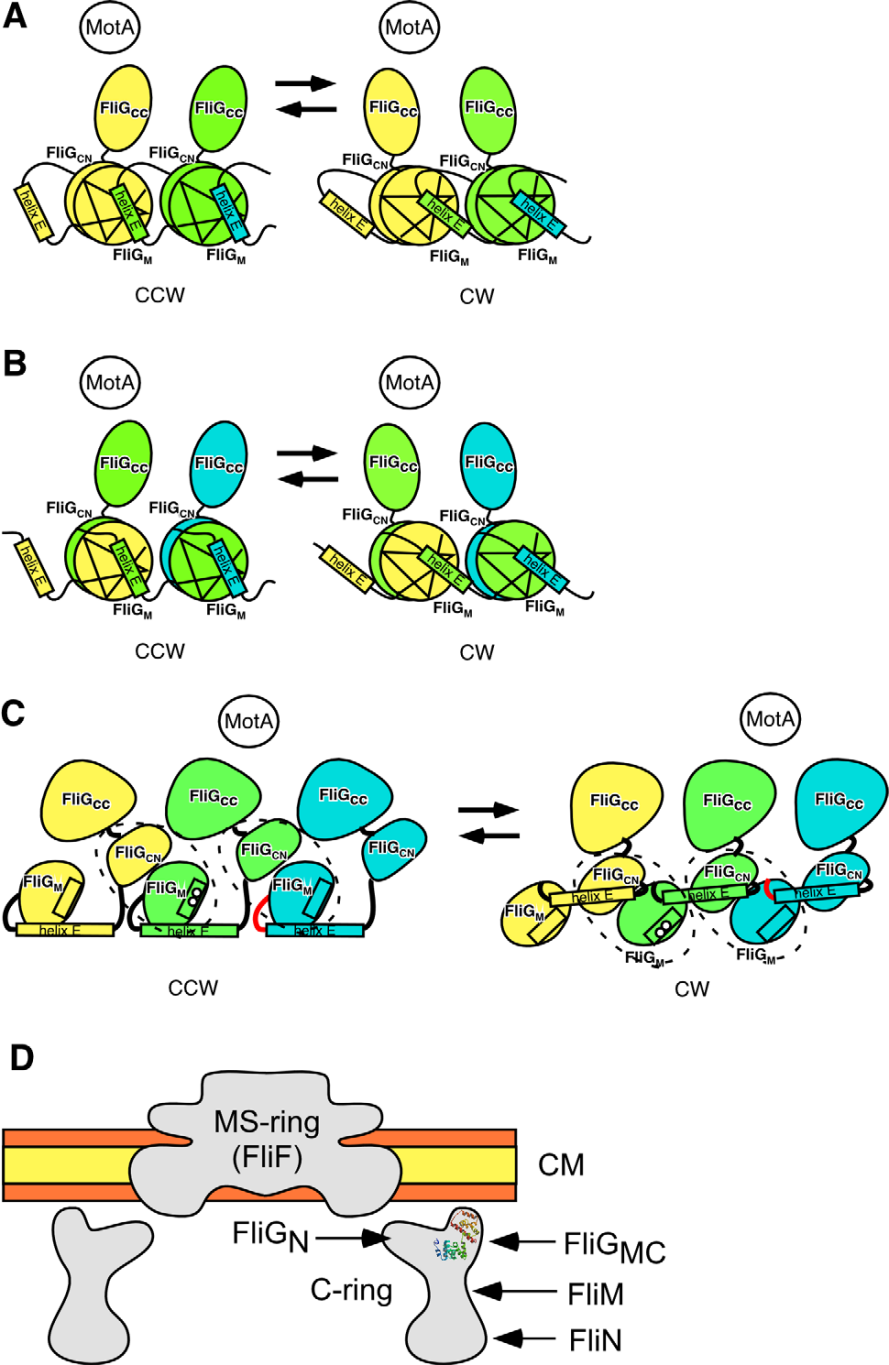
Possible models for cooperative switching. (A) The most plausible model. Two adjacent FliG molecules are colored yellow and green. The conformational change of the hinge between FliG_M_ and helix E not only changes the orientation within its own subunit but also influences the orientation of the neighboring subunit through the interaction between helix E and FliG_M_ of the neighbor. (B) Another possible model. Helix E in one subunit is linked to FliG_CN_ in the adjacent subunit. Therefore, a single functional unit consists of FliG_M_ and helix E of one molecule and FliG_CN_ and FliG_CC_ of the other molecule. Three adjacent FliG molecules are colored yellow, green, and cyan. FliG_M_ of the cyan molecule, and FliG_CN_ and FliG_CC_ of the yellow molecule are not shown. (C) The cooperative switching model proposed by Lee et al. Three FliG molecules are colored by yellow, green, and cyan. The FliG_M_-FliG_C_ units are surrounded by broken lines. The closed conformation (left panel, helix E interacts with FliG_CN_) changes to the open conformation (right panel, helix E dissociates from FliG_CN_), inducing the rotation of the FliG_M_-FliG_C_ unit and additional rotation of FliG_CN_. The box in the FliG_M_ indicates helix A. The open circles represent the sites linked to the D–E loop (colored red) by in vivo disulfide cross-linking. (D) Possible orientation of the FliG_M_-FliG_C_ unit in the rotor. The hydrophilic surface and the hydrophobic core layers of the cytoplasmic membrane are shown in orange and yellow, respectively.

FliG forms a ring on the cytoplasmic face of the MS ring [17],[18]. In vivo disulfide cross-linking experiments using Cys-substituted FliG proteins have suggested that helix A is close to the D–E loop of the adjacent FliG molecule in the FliG ring [21]. Both a conserved EHPQR motif in FliG_M_ and a conserved surface-exposed hydrophobic patch of FliG_CN_ are important for the interactions with FliM [21]. Because the conserved charged residues on helix M in FliG_CC_ are responsible for its interaction with MotA [4],[5],[25], which is embedded in the cytoplasmic membrane, helix M must lie on top of FliG_CC_ [21],[28]. Considering those facts in light of the crystal structure of Tm-FliG_MC_(ΔPEV) described here, we propose a new model for arrangement of FliG subunits in the motor (Figures 6 and 7).

In the proposed model, the conserved charged residues on helix M are located on the top of the FliG_M_-FliG_C_ unit and the EHPQR motif is present at the bottom of the unit (Figure 6B and C). The conserved hydrophobic patch, and most of the point mutation sites involved in the interaction with FliM, is localized at the bottom of the FliG_M_FliG_CN_ units around the EHPQR motif or on the interface between the FliG_M_ and FliG_CN_. The D–E loop and helix E interact with the FliG_M_ domain in the neighboring subunit, in agreement with data of in vivo cross-linking experiments, which show that residues 117 and 120 (118 and 121 in *T. martima*) on helix A of one subunit lie close to residues 166 and170 (167 and 171 in *T. martima*) on the D–E loop of the neighboring subunit [21]. In fact, these residues are very close to each other in our model in positions in which disulfide-crosslinking should occur. Moreover, the position of Cys residues that do not participate in disulfide cross-linking are far from each other in the model (Figure 6D).

Our model can also explain the results of mutational studies of CW and CCW-biased *fliG* mutants [37],[38]. The mutation sites are widely distributed from helix A to the H–I loop. Most of them are localized in three regions in our model (Figure 6A and B). In the first region, the CCW-biased mutations, which are located on helix A, affect residues close to residues targeted by CW-biased mutations, which are on a segment between helix D and E of the adjacent subunit (Figure 6A and B, 1). Because these residues are distributed on the interaction surface between the neighboring subunits, they presumably affect cooperative changes in subunit conformation. A second cluster of residues targeted by CW-biased mutations is located on the C-terminal half of helix B and the E–F loop (Figure 6A and B, 2). These mutations may change the orientation of the E–F loop and probably alter the orientation of helix E, resulting in unusual switching behavior. The third cluster of residues affected by mutations causing a CW switching bias is located near the loop between helices H and I (Figure 6A and B, 3). This region determines the relative orientation of FliG_CC_ to the FliGM-FliG_CN_ unit, and therefore the mutations may change the orientation of FliG_CC_ to cause anomalous switching behavior.

Helix E is directly involved in the switching mechanism, but how does the structure of helix E affect the orientation of the FliG_M_-FliG_C_ unit? Since the D–E loop and helix E interact with FliG_M_ in the neighboring subunit, we propose that a hinge motion of helix E may directly change the orientation of the neighboring FliG_M_ domain (Figure 7A). This mechanism could explain the cooperative switching of the motor. The conformational changes of FliM induced by association or dissociation of CheY-P may trigger conformational changes in the FliG_M_-FliG_C_ unit that it contacts, leading to a large change in the interaction between FliG_CC_ and MotA. The conformational change in one unit is probably accompanied by a conformational change in the loop between FliG_M_ and helix E. This change could influence the orientation of the neighboring subunit through the interaction between helix E and FliG_M_ of the neighbor, thereby propagating the conformational change to the neighboring subunit (Figure 7A).

If helix E actually contacts the more-distant FliG_CN_ in the crystal structure, an alternative interaction could be responsible for the cooperative switching (Figure 7B). However, the same general mechanism involving changes in the conformation of helix E would still be responsible for the cooperative switching.

Recently, Lee et al. have proposed a model for FliG arrangement and switching based on the structural differences in Aa-FliG and Tm-FliG_MC_ [28]. In the crystal structure of Aa-FliG, the hydrophobic patch in FliG_M_ is covered by the N-terminal hydrophobic residues of helix E (closed conformation), whereas the patch is exposed in Tm-FliG_MC_ (open conformation). Because mutations that may disturb the hydrophobic interaction result in strong CW-bias in motor rotation [38], the structures of Aa-FliG and Tm-FliG_MC_ are proposed to be in the CCW and CW states, respectively [28]. The hydrophobic patch is also exposed in the Tm-FliG_MC_(ΔPEV) structure, although the conformation of helix E is different from that of Tm-FliG_MC_. Since ΔPAA in *S. enterica* FliG (ΔPEV in *T. maritima*) caused an extreme CW-bias, it is possible that the dissociation of helix E from FliG_M_ leads to CW rotation. In our model, however, the hydrophobic patch of the FliG_M_ is covered by the hydrophobic residues in the C-terminal half of helix E of the adjacent subunit. This arrangement raises the possibility that the closed conformation of helix E found in the Aa-FliG structure is an artifact of crystal packing.

Lee et al. assume that the FliG_M_-FliG_C_ unit is present in the rotor ring, and hence is in agreement with the results of most of mutational studies. However, the arrangement of the subunits and the mechanism of switching are different than in our model. In their model, dynamic motion of helix E and helix n induces a large conformational change of the FliG_M_-FliG_C_ unit, including the rotation of FliG_M_-FliG_CN_ unit and relative to the FliG_CC_ to the unit, leading to a change in the arrangement of the charged residues on helix M (Figure 7C) [28]. Cooperative switching is explained by the strong interaction between FliG_CN_ of one subunit and FliG_CC_ of the adjacent subunit. However, helix A of one subunit and the D–E loop of the adjacent subunit are always at a considerable distance in both the CW and CCW states. Hence, their model cannot explain the in vivo disulfide cross-linking experiments (Figure 7C) [21]. Since our new model can explain the cross-linking data, it appears to be more plausible than the model proposed by Lee et al. [28].

Although our model is consistent with most of the previous experimental data, it still contains ambiguity. The available density map of the basal body obtained by electron cryo-microscopy is not high enough to allow fitting of the atomic model. Thus, a higher-resolution rotor-ring structure will be required to build a more precise model to explain the molecular mechanism of directional switching.

## Materials and Methods

### Bacterial Strains, Plasmids, and Media

*S. enterica* strains and plasmids used in this study are listed in Table 1. L-broth, soft agar plates, and motility media were prepared as described [39],[40]. Ampicillin was added to a final concentration of 100 µg/ml.

**Table 1:** Strains and plasmids used in this study.

Strains and Plasmids	Relevant Characteristics	Source or Reference
***Salmonella***		
SJW1103	Wild type for motility and chemotaxis	[48]
SJW46	*fliC*(Δ204–292)	[49]
SJW2811	*fliG*(ΔPAA)	[10]
SJW3076	Δ(*cheA–cheZ*)	[30]
MKM1	Δ*fliG*	[19]
MM3076iC	Δ(*cheA–cheZ*), *fliC* (Δ204–292)	[50]
MMG1001	Δ*fliG fliC*(Δ204–292)	This study
**Plasmids**		
pET19b	Expression vector	Novagen
pTrc99A	Expression vector	Pharmacia
pGKM3000	pET19b/His-FliG	[19]
pGKM4000	pET19b/His-FliG(ΔPAA)	This study
pGMM3500	pTrc99A/His-FliG	This study
pGMM4500	pTrc99A/His-FliG(ΔPAA)	This study
pGMM5000	pET22b/Tm-FliG_MC_(ΔPEV)	This study

### Motility Assay

Fresh colonies were inoculated on soft tryptone agar plates and incubated at 30°C.

### Bead Assay for Motor Rotation

Bead assays were carried out using polystyrene beads with diameters of 0.8, 1.0, and 1.5 mm (Invitrogen), as described before [8]. Torque calculation was carried out as described [8].

### Preparation of Whole Cell Proteins and Immunoblotting

Cultures of *S. enterica* cells grown at 30°C were centrifuged to obtain cell pellets. The cell pellets were resuspended in SDS-loading buffer, normalized in cell density to give a constant amount of cells. Immunoblotting with polyclonal anti-FliG antibody was carried out as described [41].

### Purification of His-FliG and His-FliG(ΔPAA) and Limited Proteolysis

His-FliG and His-FliG(ΔPAA) were purified by Ni-NTA affinity chromatography as described before [39]. His-FliG and its mutant variant (0.5 mg/ml) were incubated with trypsin (Roche Diagnostics) at a protein to protease ratio of 300∶1 (w/w) in 50 mM K_2_HPO_4_-NaH_2_PO_4_ pH 7.4 at room temperature. Aliquots were collected at 0, 5, 15, 30, 60, 90, and 120 min and trichloroacetic acid was added to a final concentration of 10%. Molecular mass of proteolytic cleavage products was analyzed by a mass spectrometer (Voyager DE/PRO, Applied Biosystems) as described [42]. N-terminal amino acid sequence was done as described before [42].

### Purification, Crystallization, Data Collection, and Structure Determination of Tm-FliG_MC_(ΔPEV)

Tm-FliG_MC_(ΔPEV) was purified as described previously [23]. Crystals of Tm-FliG_MC_(ΔPEV) were grown at 4°C using the hanging-drop vapor-diffusion method by mixing 1 µl of protein solution with 1 µl of reservoir solution containing 0.1 M sodium phosphate-citrate buffer pH 4.2–4.4, 36%–50% PEG200, and 200 mM NaCl. Initially, we tried to solve the structure by the molecular replacement method using Tm-FliG_MC_ structure (PDB ID: 1 lkv) as a search model. However, no significant solution was obtained, even though individual domains were used as search models. Therefore, we prepared heavy-atom derivative crystals and determined the structure using the anomalous diffraction data from the derivatives.

Derivative crystals were prepared by soaking in a reservoir solution containing K_2_OsCl_6_ at 50% (v/v) saturation for one day. Crystals of Tm-FliG_MC_(ΔPEV) and its Os derivatives were soaked in a solution containing 90%(v/v) of the reservoir solution and 10%(v/v) 2-Methyl-2,4-pentanediol for a few seconds, then immediately transferred into liquid nitrogen for freezing. All the X-ray diffraction data were collected at 100 K under nitrogen gas flow at the synchrotron beamline BL41XU of SPring-8 (Harima, Japan), with the approval of the Japan Synchrotron Radiation Research Institute (JASRI) (Proposal No. 2007B2049). The data were processed with MOSFLM [43] and scaled with SCALA [44]. Phase calculation was performed with SOLVE [45] using the anomalous diffraction data from Os-derivative crystals. The best electron-density map was obtained from MAD phases followed by density modification with DM [44]. The model was constructed with Coot [46] and was refined against the native crystal data to 2.3 Å using the program CNS [47]. About 5% of the data were excluded from the data for the R-free calculation. During the refinement process, iterative manual modifications were performed using “omit map.” Data collection and refinement statistics are summarized in Tables S1 and S2, respectively.

## Supporting Information

Figure S1 Effects of the in-frame deletion of residues PAA of *S. enterica* FliG on the direction of flagellar motor rotation and torque generation. (A) Measurement of CCW and CW rotation of the flagellar motor. Rotation individual flagellar motors of SJW46 transformed with pGMK3000 (pET19b/His-FliG, indicated as WT) (left) or pGMK3000 (pET19b/His-FliG(ΔPAA), indicated as FliG(ΔPAA)) (right) were carried out by tracking the position of 1.0 µm bead attached to the sticky flagellar filament. Measurements were made at ca. 23°C. CCW, counterclockwise rotation; CW, clockwise rotation. (B) Measurements of the rotational speeds of single flagellar motors labeled with 0.8 µm (right), 1.0 µm (left), and 1.5 µm (middle) beads.
(0.06 MB TIF)

Figure S2 Motility assays for complementation of the motility of a Δ*fliG* null mutant (left) and a *fliG*(ΔPAA) mutant transformed with pET19b (indicated as Low-V), pTrc99A (indicated as High-V), pGMK4000 (pET19b/His-FliG(ΔPAA), indicated as Low-FliG(ΔPAA)), and pGMM4500 (pTrc99A/His-His-FliG(ΔPAA), indicated as High-FliG(ΔPAA)) in semi-solid agar. The plates were incubated at 30°C for the length of time indicated.
(0.31 MB TIF)

Figure S3 Molecular packing in the crystal. (A) Stereo view of the molecular packing of Tm-FliG_MC_(ΔPEV) in the *P6_2_* crystal, projected down the c axis. Molecules are indicated by Cα backbone traces. A pair of FliG molecules related by two-fold crystallographic symmetry is highlighted in cyan and yellow for FliG_M_ and FliG_C_, respectively. Other molecules are shown in grey. G186 and V195 are indicated by blue and magenta balls, respectively. G186 can be connected to V195 (solid line) or V195' (dashed line). (B) Stereo view of four symmetry-related molecules of Tm-FliG_MC_ that form the inter-molecular four-helix bundle structure in the *P6_4_22* crystal (PDB code: 1lkv). FliG_M_ and FliG_CN_ of the subunit colored by cyan form the FliG_M_-FliG_CN_ units with FliG_CN_ and FliG_M_ of the subunit colored by yellow, respectively, and FliG_M_ and FliG_CN_ of the subunit colored by green form the FliG_M_-FliG_CN_ units with FliG_CN_ and FliG_M_ of the subunit colored by orange, respectively. (C) Stereo view of the molecular packing of Aa-FliG in the *P2_1_* crystal (PDB code: 3hjl), projected down the c axis. The molecules related by crystallographic *2_1_* symmetry are colored by cyan and yellow. The cyan molecule located in the centre of the panel is labeled, and helix n and helix E of the center molecule are highlighted in orange.
(2.96 MB TIF)

Table S1 Data collection statistics.
(0.04 MB PDF)

Table S2 Refinement statistics.
(0.03 MB PDF)
