# Supplementary figures and images for: Structural Insight into the Rotational Switching Mechanism of the Bacterial Flagellar Motor
Source: PLoS Biol. 2011 May 10;9(5):e1000616. doi: 10.1371/journal.pbio.1000616 (PMC3091841; doi:10.1371/journal.pbio.1000616)

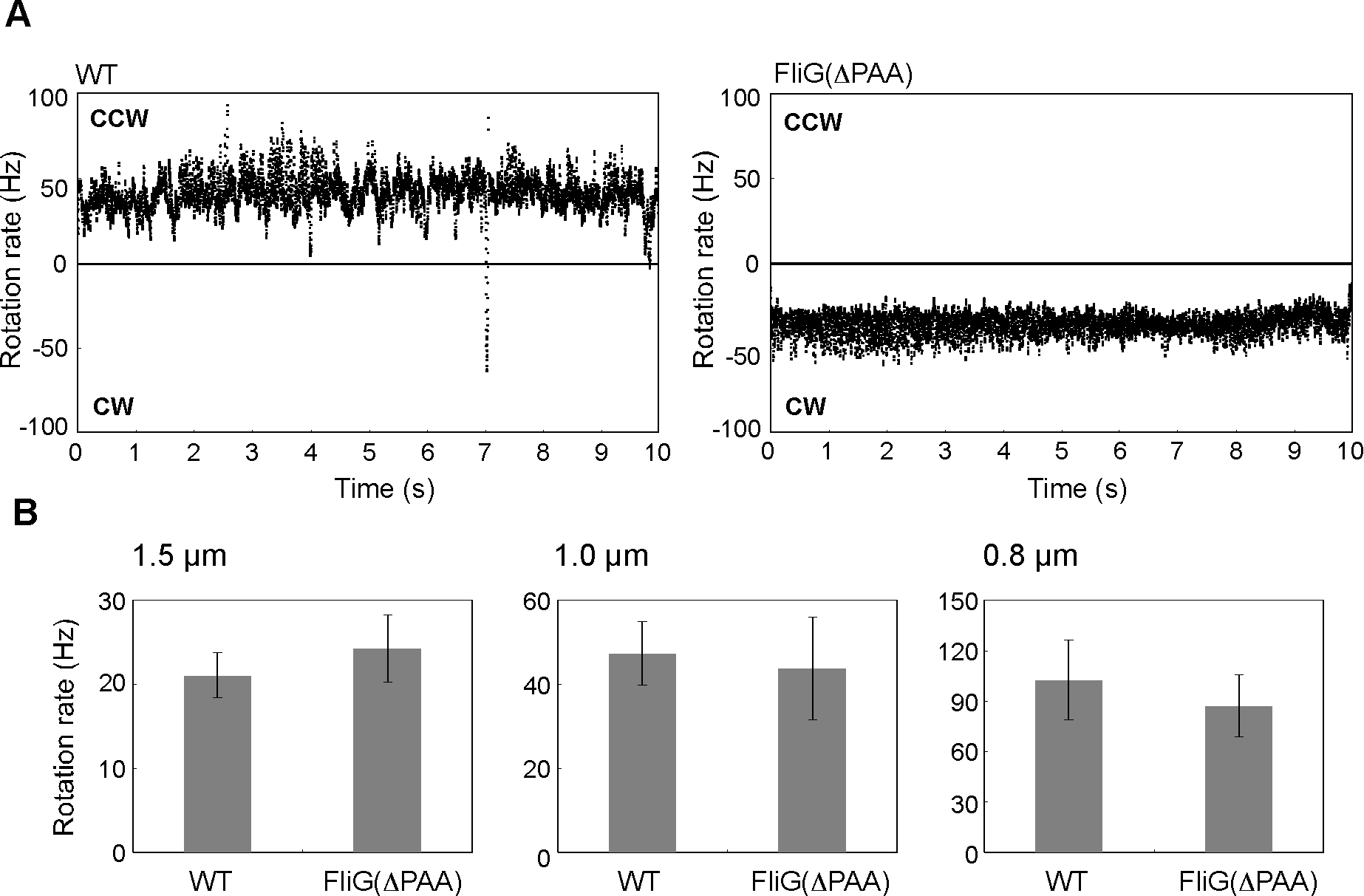

Supplement: Figure S1 — Effects of the in-frame deletion of residues PAA of S. enterica FliG on the direction of flagellar motor rotation and torque generation. (A) Measurement of CCW and CW rotation of the flagellar motor. Rotation individual flagellar motors of SJW46 transformed with pGMK3000 (pET19b/His-FliG, indicated as WT) (left) or pGMK3000 (pET19b/His-FliG(ΔPAA), indicated as FliG(ΔPAA)) (right) were carried out by tracking the position of 1.0 µm bead attached to the sticky flagellar filament. Measurements were made at ca. 23°C. CCW, counterclockwise rotation; CW, clockwise rotation. (B) Measurements of the rotational speeds of single flagellar motors labeled with 0.8 µm (right), 1.0 µm (left), and 1.5 µm (middle) beads. (0.06 MB TIF) [file pbio.1000616.s001.tif]

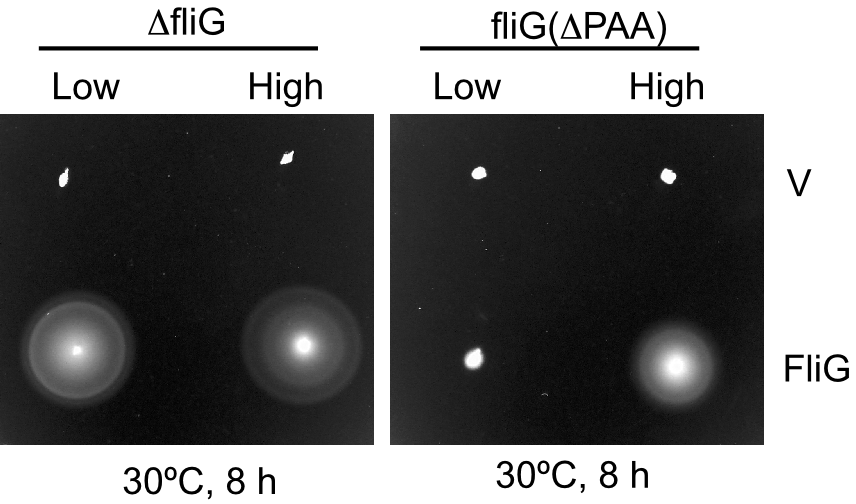

Supplement: Figure S2 — Motility assays for complementation of the motility of a ΔfliG null mutant (left) and a fliG(ΔPAA) mutant transformed with pET19b (indicated as Low-V), pTrc99A (indicated as High-V), pGMK4000 (pET19b/His-FliG(ΔPAA), indicated as Low-FliG(ΔPAA)), and pGMM4500 (pTrc99A/His-His-FliG(ΔPAA), indicated as High-FliG(ΔPAA)) in semi-solid agar. The plates were incubated at 30°C for the length of time indicated. (0.31 MB TIF) [file pbio.1000616.s002.tif]

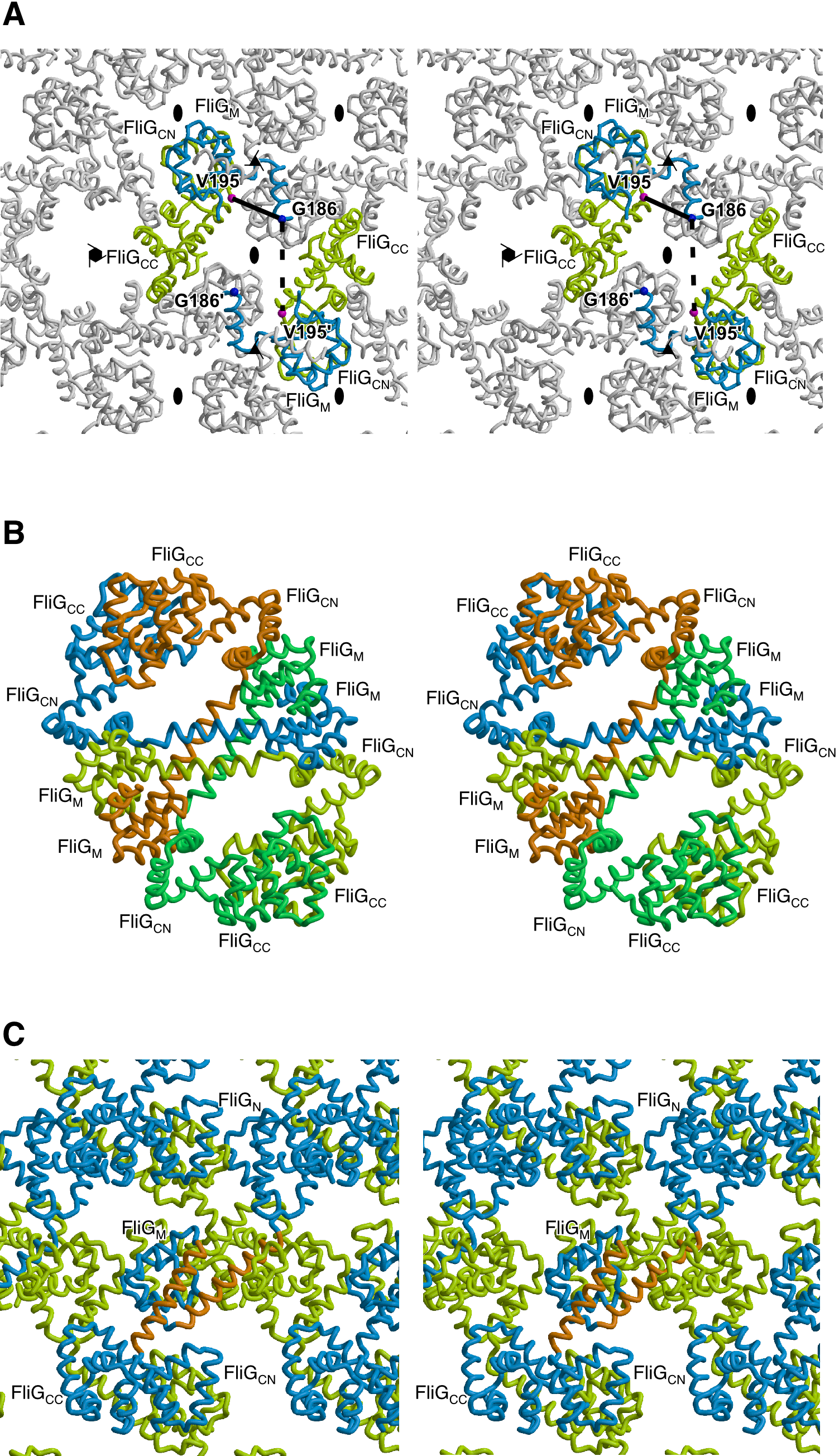

Supplement: Figure S3 — Molecular packing in the crystal. (A) Stereo view of the molecular packing of Tm-FliGMC(ΔPEV) in the P62 crystal, projected down the c axis. Molecules are indicated by Cα backbone traces. A pair of FliG molecules related by two-fold crystallographic symmetry is highlighted in cyan and yellow for FliGM and FliGC, respectively. Other molecules are shown in grey. G186 and V195 are indicated by blue and magenta balls, respectively. G186 can be connected to V195 (solid line) or V195' (dashed line). (B) Stereo view of four symmetry-related molecules of Tm-FliGMC that form the inter-molecular four-helix bundle structure in the P6422 crystal (PDB code: 1lkv). FliGM and FliGCN of the subunit colored by cyan form the FliGM-FliGCN units with FliGCN and FliGM of the subunit colored by yellow, respectively, and FliGM and FliGCN of the subunit colored by green form the FliGM-FliGCN units with FliGCN and FliGM of the subunit colored by orange, respectively. (C) Stereo view of the molecular packing of Aa-FliG in the P21 crystal (PDB code: 3hjl), projected down the c axis. The molecules related by crystallographic 21 symmetry are colored by cyan and yellow. The cyan molecule located in the centre of the panel is labeled, and helix n and helix E of the center molecule are highlighted in orange. (2.96 MB TIF) [file pbio.1000616.s003.tif]
