# Supplementary material for: Structural Insight into the Rotational Switching Mechanism of the Bacterial Flagellar Motor
Source: PLoS Biol. 2011 May 10;9(5):e1000616. doi: 10.1371/journal.pbio.1000616 (PMC3091841; doi:10.1371/journal.pbio.1000616)
Supplement: Table S1 — Data collection statistics. (0.04 MB PDF) [file pbio.1000616.s004.pdf]

	Native	Os-derivative
Space group	$P6_2$	
Cell dimensions		
a, b, c (Å)	93.15, 93.15, 48.39	92.47, 92.47, 47.81
α, β, γ (°)	90, 90, 120	90, 90, 120
Wavelength (Å)	1.00000	1.13987
Resolution (Å)	42.0-2.3 (2.42-2.3)	48.0-3.2 (3.37-3.2)
R_{merge}	6.5 (33.6)	10.2 (37.0)
I/σI	21.3 (5.3)	17.1 (6.2)
Completeness (%)	97.9 (97.9)	99.7 (99.7)
Redundancy	6.2 (6.3)	5.8 (6.0)
