# Supplementary material for: Structural Insight into the Rotational Switching Mechanism of the Bacterial Flagellar Motor
Source: PLoS Biol. 2011 May 10;9(5):e1000616. doi: 10.1371/journal.pbio.1000616 (PMC3091841; doi:10.1371/journal.pbio.1000616)
Supplement: Table S2 — Refinement statistics. (0.03 MB PDF) [file pbio.1000616.s005.pdf]

Resolution (Å)	33.6-2.3 (2.44-2.3)
R_{work} /R_{free}	22.4/23.9 (25.1/30.4)
No. atoms	
Protein	1679
Water	114
B-factors	
Protein	48.8
Water	65.9
R.m.s. deviations	
Bond lengths (Å)	0.008
Bond angles (°)	1.3
Ramachandran plot (%)	
Most favored	96.4
Additionally	3.6
allowed	0
Generously allowed	0
Disallowed	
